# Gastrointestinal Disorder Associated with Olmesartan Mimics Autoimmune Enteropathy

**DOI:** 10.1371/journal.pone.0125024

**Published:** 2015-06-23

**Authors:** Sophie Scialom, Georgia Malamut, Bertrand Meresse, Nicolas Guegan, Nicole Brousse, Virginie Verkarre, Coralie Derrieux, Elizabeth Macintyre, Philippe Seksik, Guillaume Savoye, Guillaume Cadiot, Lucine Vuitton, Lysiane Marthey, Franck Carbonnel, Nadine Cerf-Bensussan, Christophe Cellier

**Affiliations:** 1 Université Paris Descartes-Sorbonne Paris Centre, Paris, France; 2 Gastroenterology department, Hôpital Européen Georges Pompidou APHP, Paris, France; 3 Laboratory of Intestinal Immunology, Inserm UMR 1163 and Institute Imagine, Paris, France; 4 Pathology department, Hôpital Necker Enfants Malades APHP, Paris, France; 5 Biological Hematology, Hôpital Necker Enfants Malades APHP, Paris, France; 6 Gastroenterology department, Hôpital Saint Antoine APHP, Paris, France; 7 Gastroenterology, CHU Rouen, Rouen, France; 8 Gastroenterology department, CHU Reims, Reims, France; 9 Gastroenterology department, CHRU Besançon, Besançon, France; 10 Gastroenterology department Hôpital Bicêtre APHP, Paris, France; University of Southampton School of Medicine, UNITED KINGDOM

## Abstract

**Background and Objectives:**

Anti-hypertensive treatment with the angiotensin II receptor antagonist olmesartan is a rare cause of severe Sprue-like enteropathy. To substantiate the hypothesis that olmesartan interferes with gut immune homeostasis, clinical, histopathological and immune features were compared in olmesartan-induced-enteropathy (OIE) and in autoimmune enteropathy (AIE).

**Methods:**

Medical files of seven patients with OIE and 4 patients with AIE enrolled during the same period were retrospectively reviewed. Intestinal biopsies were collected for central histopathological review, T cell Receptor clonality and flow cytometric analysis of isolated intestinal lymphocytes.

**Results:**

Among seven olmesartan-treated patients who developed villous atrophy refractory to a gluten free diet, three had extra-intestinal autoimmune diseases, two had antibodies reacting with the 75 kilodalton antigen characteristic of AIE and one had serum anti-goblet cell antibodies. Small intestinal lesions and signs of intestinal lymphocyte activation were thus reminiscent of the four cases of AIE diagnosed during the same period. Before olmesartan discontinuation, remission was induced in all patients (7/7) by immunosuppressive drugs. After interruption of both olmesartan and immunosuppressive drugs in six patients, remission was maintained in 4 but anti-TNF-α therapy was needed in two.

**Conclusion:**

This case-series shows that olmesartan can induce intestinal damage mimicking AIE. OIE usually resolved after olmesartan interruption but immunosuppressive drugs may be necessary to achieve remission. Our data sustain the hypothesis that olmesartan interferes with intestinal immuno regulation in predisposed individuals.

## Introduction

Olmesartan is an angiotensin II receptor antagonist used to treat arterial hypertension. Several cases of chronic diarrhea with weight loss, anaemia and low serum albuminemia have been reported after the use of olmesartan [1]. The Mayo Clinic was the first to report 22 cases of severe Sprue-like enteropathy associated with olmesartan [2]. All patients displayed villous atrophy and 14/22 had intraepithelial hyperlymphocytosis. Together with the high frequency of HLA-DQ2 genotype (present in 68%), these features were reminiscent of celiac disease. However no serum anti-transglutaminase antibodies (in absence of IgA and IgG deficiency) were detected and none of the patients responded to a gluten-free diet. Notably, three of them had detectable serum anti-enterocyte antibodies. In another series of 72 patients with unexplained intestinal villous atrophy and negative celiac serology, 16 cases were ascribed to the use of olmesartan [3]. More recently a French National cohort study reported 36 cases of olmesartan-induced enteropathy (OIE), 32/36 of which had villous atrophy. None had serum anti-transglutaminase or anti-enterocyte antibodies but 9/11 had anti-nuclear antibodies [4]. The mechanisms underlying duodenal villous atrophy and intraepithelial hyperlymphocytosis (65% of cases) [1], remain elusive. Cell-immunity mediated damage is suggested by the long delay between the onset of olmesartan therapy and the development of enteropathy with diarrhea [2]. The present examination of clinico-pathological features and phenotypic characterization of intestinal lymphocytes in seven patients with OIE eliminated other causes of severe enteropathies, notably common variable immunodeficiency and refractory celiac disease but revealed striking similarities with four cases of adult autoimmune enteropathy (AIE) referred to our institution during the same period. Moreover all OIE patients responded to immunosupressive drugs before olmesartan discontinuation and anti-TNF- therapy was necessary to maintain or achieve remission in 2 out of 6 patients after olmesartan interruption.

## Methods

### Patients

Medical files of patients treated with olmesartan and presenting severe enteropathy (patients 1–7) and of patients with autoimmune enteropathy (AIE) (patients 8–11) were reviewed retrospectively. Patients were followed-up until June 2014. Onset of lymphoma in AIE patient 9 and patient 10 was previously reported [5, 6].

### Material & Methods

Clinical data recorded for each patient included age, sex, symptoms, and body mass index (BMI). Presence of anti-AIE-75KD antibodies, anti-nuclear and anti-tissue antibodies (anti-mitochondria, -LKM1, -smooth muscle, -thyroid) and serological tests of celiac disease (serum immunoglobulin IgA (AGA) and IgG (AGG) anti-gliadin antibodies, serum IgA class endomysial antibodies (EMA), serum anti-human tissue transglutaminase IgA (tTG) antibodies) were also recorded. HLA-DRB1 and DQB1 genotyping was performed by hybridization with sequence-specific oligonucleotides following amplification by PCR, using the InnoLipa HLA genotyping test (Abott, Rungis France) [7]. Endoscopic evaluation included upper gastrointestinal endoscopy or enteroscopy with gastric and small intestinal biopsies, colonoscopy with colonic biopsies. Clinical response was defined by a reduction of 50% of stool frequency and recovery of at least 50% of body weight loss. Mucosal response was defined by total or partial recovery of a normal villous epithelium [8].

For histological analysis, gastrointestinal specimens were fixed in 10% formalin, embedded in paraffin, and 5 μm sections stained with H&E and Giemsa. Villous atrophy was assessed on two to 3 duodenal biopsies as described [9]. Duodenal lymphocytosis was defined by counts of intraepithelial lymphocytes (IEL) over 30 per 100 duodenal epithelial cells (EC), lymphocytic gastritis by IEL counts over 25 per 100 gastric columnar EC and lymphocytic colitis by IEL counts over 20 per 100 surface colonic EC. Apoptotic bodies (single-cell karyorrhectic debris) were assessed and were enumerated per 10 crypts [9].

Isolation of IEL, lamina propria lymphocytes (LPL) and peripheral blood lymphocytes (PBL) was performed as described [8, 10, 11]. Surface Lymphocyte phenotype was assessed by 8-color flow-cytometry. Briefly, pellets of 2-5x10^4^ cells were incubated for 20 minutes with mixof antibodies directly conjugated with FITC, APC, PE, PE/Cy7 BD-Horizon-V450, PerCP/Cy5.5, AmCyan, APC-H7 at optimal concentrations. The following antibodies were used: CD45, CD3, CD4, CD8, NKP46, CD56, CD57, NKG2A, control isotypes (BD Biosciences, Le Pont de Claix, France), CD103, NKG2C, CD94, TCRαβ, TCRγδ (Beckman Coulter, Nyon, Switzerland) and NKG2C (R&D system, Abingdon, UK). For intracellular FOXP3 detection, cells were surface labeled with CD45, CD4 and CD25, and then fixed, permeabilized using Human FOXP3 Buffer Set (BD Biosciences) and labelled with PE-conjugated anti-human or control isotype (BD Biosciences). Fluorescence staining was analyzed with a FACSCanto II flow cytometer using the DIVA software (BD Biosciences) and gated on CD45^+^ cells.

Molecular detection of clonal TCRγ chains rearrangements was performed on DNA extracted from frozen specimens and from PBL by multiplex PCR and confirmed by analysis of heteroduplex formation, as described [8].

### Ethics Statement

The study was approved by the Ile-de-France II ethical committee (Paris, France).

## Results

### Clinical and immunological characteristics (Table 1)

**Table 1:** Clinical and immune characteristics.

Case	Sex	Age (y)	Autoimmunediseases	BMI	DQ2/8	Anti AIE 75 kDa	anti-E	tTG	IGA	ANA	Duod	Lymphocytosis		
												Duod	Sto	Col
**Olme-sartan**														
**1**	F	74	Goujerot Sjogren	17	+	+	*nd*	+	-	+	TVA	30%	-	-
**2**	F	72	-	23	*nd*	+	*nd*	-	-	*nd*	STVA	40%	-	-
**3**	F	69	Uveitis Cholangitis	17	+	-	-	-	*nd*	+	STVA	40%	-	-
**4**	M	79	-	20	+	-	-	-	*nd*	+	TVA	30%	-	-
**5**	M	60	-	21	-	-	-	-	*nd*	-	TVA	100%	+	-
**6**	F	65	Cholangitis	20	-	-	*nd*	-	-	+	TVA	30%	-	-
**7**	M	77	-	24	+	*nd*	-*	-	-	*nd*	STVA	30%	-	-
**Mean**		**71**		**20**	**67%**	**33%**	**0%**	**14%**	**0%**	**80%**		**43%**	**14%**	**0**
**AIE**														
**8**	F	17	Auto I Pancreatitis anti-phospholipid Sd Polyarthritis	16	+	+	*nd*	-	-	+	TVA	40%	-	-
**9**	F	23	-	21	-	+	+	-	-	+	TVA	90%	+	+
**10**	F	19	-	20	-	+	-	+	-	*nd*	STVA	57%	-	+
**11**	F	41	-	18	-	+	*nd*	+	+	+	STVA	65%	-	-
**Mean**		**25**	**25%**	**19**	**25%**	**100%**	**50%**	**50%**	**25%**	**100%**		**63%**	**25%**	**50%**

*: detection of serum anti-goblet cells antibodies.

Ab: antibody. Anti-E: anti-enterocyte Ab. ANA: anti-nuclear Ab. BMI: Body Mass Index. Col: colon. Duod: duodenum. EMA: IgA anti-endomysium. IGA: IgA anti-gliadin. Lymphocytosis: number of intraepithelial lymphocytes for 100 epithelial cells. LC: lymphocytic colitis. LG: lymphocytic gastritis. Sto: stomach. tTG: IgA anti-transglutaminase. VA: villousatrophy. TVA: total villousatrophy. ST VA: sub-totalvillousatrophy. PVA: partial villousatrophy. y: years. Noserum anti-tTG IgG or antigliadin IgG was found. No IgA anti-endomysium was found.

Seven patients treated by olmesartan were referred to our medical center between 2000 and 2014 for unexplained severe enteropathy with villous atrophy refractory to a gluten free diet (Table 1). All had chronic diarrhea with malnutrition. Four of them (patients 1, 2, 4 and 5) were treated by parenteral nutrition at time of admission and two patients had already been hospitalized for severe hypokalemia (patients 1 and 4). Three patients had extra-intestinal autoimmune diseases. Four patients had the celiac HLA-DQ2/DQ8 susceptibility genotype. Celiac antibodies (IgA and IgG anti-gliadin and anti-transglutaminase, IgA anti-endomysium) were tested before initiating a gluten free diet and were negative except in one HLA-DQ2 patient (patient 1) who had low titers of serum IgA anti-transglutaminase (Table 1). Primary immunoglobulin deficiency was eliminated in all patients. Serum antibodies reacting with goblet cells or with the brush border 75-kilodalton antigen (AIE-75KD) were detected in one (1/4) and 2 patients (2/6), respectively. Serum anti-nuclear antibodies were detected in 4 patients (4/5).

### Histopathological findings (Fig 1)

**Fig 1:**
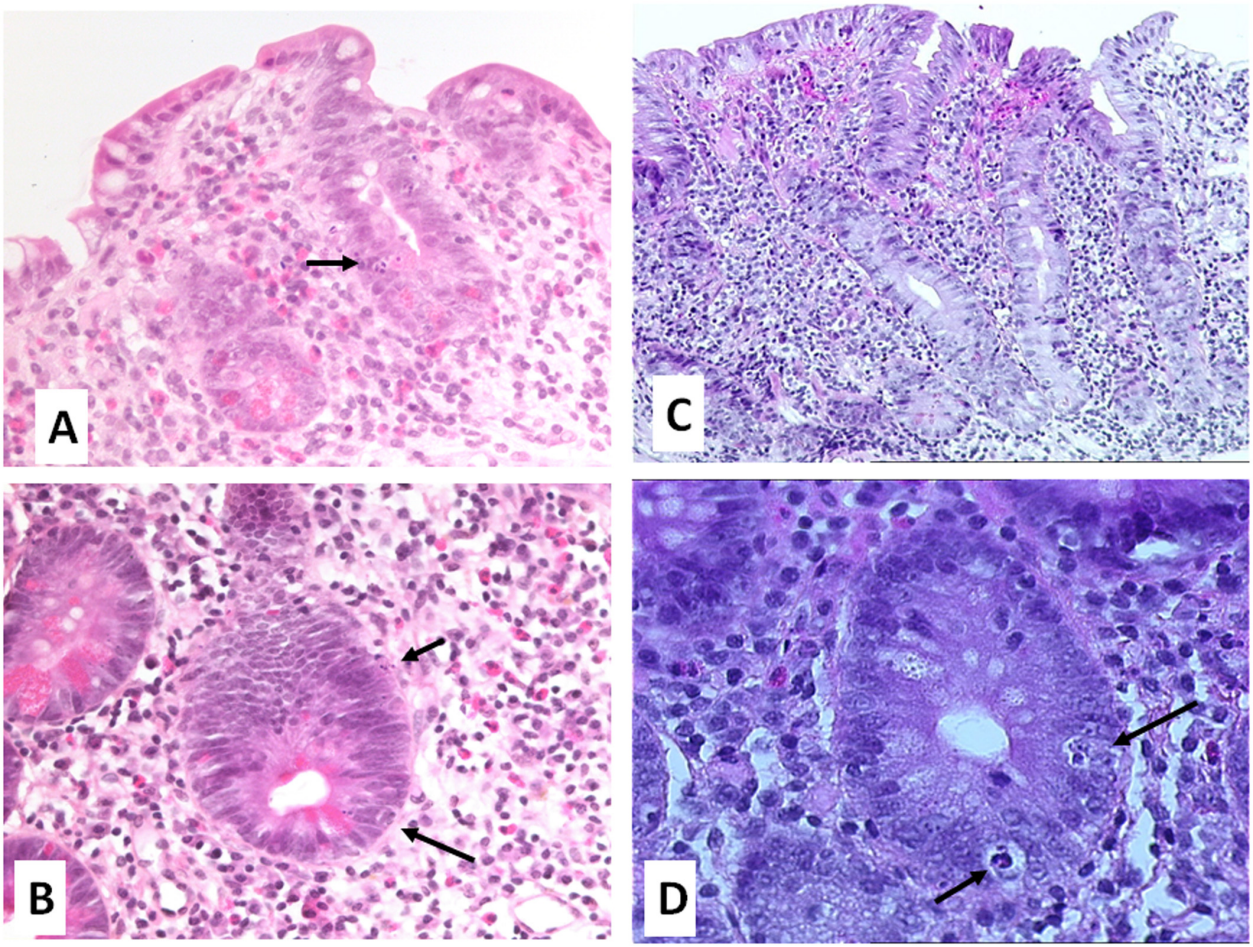
H&E staining of the duodenal biopsies of one patient treated with Olmesartan (patient 2) (A, B) and of one patient with AIE (patient 7) (C, D) showing subtotal villous atrophy (A, C: original magnification x 100) with glandular apoptosis (B, D: original magnification x200).

Duodenal or jejunal biopsies showed subtotal (patients 2, 3 and 7) or total villous atrophy (patients 1,4, 5 and 6), dense lymphocyte and plasma cell *lamina propria* infiltration and crypt rarefaction (patients 1–7), paucity of goblet cells and glandular apoptosis (patients 2 and 4). Such intestinal pathological features were reminiscent of those observed in 4 patients with adult autoimmune enteropathy (AIE) investigated during the same period (Fig 1). An intestinal collagenous subepithelial layer thicker than 10μm was observed in patient 1, as in one AIE patient (patient 9). Patient 4 had very high numbers of IEL in the duodenum (100%) and displayed lymphocytic gastritis. Similarly, two AIE patients (9 and 10) with increased numbers of duodenal IEL also had lymphocytic gastritis and/or colitis (Table 1).

### Lymphocyte isolation and flow cytometry analysis (Tables 2, 3 and 4)

**Table 2:** Phenotype of intestinal intraepithelial lymphocytes.

Case	CD103+		CD8+	CD4+	TCR		CD94+	CD56+	CD57+	NKP46	NKG2A	NKG2C
	CD3+	CD3-			αβ	γδ						
**Olmesartan**												
**1**	71%	2%	67%	30%	86%	5%	20%	-	3%	3%	5%	5%
**2**	60%	21%	63%	23%	74%	3%	-	44%	-	26%	-	10%
**3**	69%	23%	64%	11%	59%	12%	27%	37%	16%	37%	17%	3%
**4**	83%	5%	66%	35%	92%	2%	46%	5%	18%	6%	5%	8%
**5**	47%	30%	54%	19%	64%	6%	20%	10%	24%	35%	7%	80%
**6**	53%	14%	46%	29%	72%	4%	16%	11%	9%	12%	10%	7%
**Median**	**65%**	**17%**	**64%**	**26%**	**73%**	**4%**	**20%**	**11%**	**16%**	**19%**	**7%**	**7%**
**AIE**												
**8**	71%	5%	70%	24%	85%	2%	64%	22%	2%	6%	5%	80%
**9**	79%	1%	80%	4%	95%	3%	27%	3%	10%	-	-	-
**10**	88%	4%	90%	4%	94%	0%	8%	6%	7%	1%	-	-
**11**	93%	3%	40%	60%*	88%	8%	13%	2%	1%	7%	4%	5%
**Median**	**84%**	**4%**	**75%**	**14%**	**91%**	**3%**	**20%**	**5%**	**5%**	**6%**	**4%**	**48%**
**Normal value (%)**	**80–95**	**2–20**	**60–85**	**5–15**	**70–88**	**12–20**	**16–38**	**9–19**	**0**	**5–15**	**11–38**	**1–5**

*: excess of CD4+ IEL with onset of CD4 lymphoma after two years treatment with azathioprine (Case published in Malamut et al, ClinGastHepatol 2014); flow cytometry analysis of AIE onset is not available.

**Table 3:** Phenotype of lamina propria intestinal lymphocytes.

Case	CD103+ ****CD3+****	CD103- CD3+	CD8+	CD4+	TCR alpha beta	CD19+	CD94+	CD56+	CD57+	NKG2C	CD4+ CD25+ FOXP3+
**Olmesartan**											
**1**	34%	52%	35%	53%	80%	8%	3%	-	1%	3%	5%
**2**	25%	53%	50%	45%	74%	-	-	42%	-	6%	6%
**3**	28%	62%	39%	51%	84%	8%	12%	5%	10%	-	6%
**4**	23%	66%	38%	56%	85%	8%	17%	1%	11%	1%	11%
**5**	42%	39%	47%	38%	73%	8%	3%	5%	30%	17%	4%
**6**	26%	52%	37%	39%	68%	23%	10%	2%	9%	5%	10%
**Median**	**27%**	**53%**	**39%**	**48%**	**77%**	**8%**	**10%**	**5%**	**10%**	**5%**	**6%**
**AIE**											
**8**	23%	49%	40%	38%	73%	18%	24%	14%	5%	3%	8%
**9**	54%	45%	55%	38%	-	-	-	-	-	-	4%
**10**	38%	57%	47%	49%	94%	5%	7%	6%	8%	-	8%
**11**	38%	75%	33%	54%	83%	1%	27%	10%	5%	16%	1%
**Median**	**38%**	**53%**	**43%**	**44%**	**83%**	**5%**	**24%**	**10%**	**5%**	**10%**	**6%**
**Normal Value (%)**	**20–35**	**40–70**	**30–50**	**40–60**	**65–85**	**5–10**	**<8**	**<10**	**<1**	**<0,5**	**2–12**

**Table 4:** Phenotype of peripheral blood lymphocytes.

Case	CD103+ CD3-	CD3+ CD8+	CD3+ CD4+	TCR alpha beta	CD19+	CD94+	CD56+	CD57+	NKG2C	LT CD4+ CD25+ FOXP3+
**Olmesartan**										
**1**	0.5%	10%	49%	-	11%	-	-	-	-	3%
**2**	0.5%	8%	63%	69%	-	-	52%	-	-	5%
**3**	0.1%	25%	50%	71%	17%	15%	13%	16%	-	3%
**4**	0.1%	13%	62%	-	30%	-	-	-	1%	4%
**5**	-	19%	47%	-	6%	-	-	-	6%	6%
**6**	0.1%	37%	39%	81%	8%	-	8%	16%	0%	1%
**Median**	**0.1%**	**16%**	**50%**	**71%**	**11%**	**15%**	**13%**	**16%**	**1%**	**4%**
**AIE**										
**8**	0.0%	34%	39%	68%	7%	23%	57%	31%	73%	4%
**9**	0.4%	38%	50%	-	-	30%	20%	26%	-	-
**10**	0.0%	30%	61%	87%	4%	6%	11%	19%	-	4%
**11**	0.2%	22%	61%	78%	1%	12%	5%	13%	3%	3%
**Median**	**0.1%**	**32%**	**55%**	**78%**	**4%**	**17%**	**16%**	**23%**	**38%**	**4%**
**Normal Value (%)**	**<1**	**30–50**	**40–60**	**65–85**	**5–20**	**5–25**	**5–25**	**5–25**	**0**	**2–12**

Yield of isolated IEL was low or normal in all patients with OIE and AIE (0.1–0,3x10^6^/6 duodenal biopsies) except for patient 11 with AIE complicated by a CD4^+^ lymphoma which infiltrated the gut epithelium. In contrast, and in keeping with *lamina propria* infiltration in tissue sections, the yield of LPL was increased (0,8 à 1.5x10^6^/ 6 duodenal biopsies, normal 0.5±0.1). Flow cytometry analysis showed a predominance of CD3+ TCRαβ+T cells in IEL and LPL. In contrast with observations in celiac disease, the frequency of γδT IEL was low in both OIE and AIE, while the frequency of CD4+ IEL was often high in OIE. A very high count of CD4+ IEL cells was observed in patient 10, who had AIE complicated by a CD4+small cell T lymphoma which infiltrated the epithelium. In some OIE and AIE patients, T cells expressing the CD57 or NKG2C NK markers were increased in epithelium and *lamina propria*, suggesting chronic activation. Interestingly, the frequency of the normal subset of IEL lacking surface CD3 and expressing the NK marker NKP46 was higher in patients with OIE than with AIE (Table 2), even if the difference was not statistically significant. The frequency of CD3- IEL remained however within normal values, thus eliminating type II refractory CD (Tables 2 and 3) [10]. No deficit in the number of intestinal and blood regulatory T cells CD4+CD25+FOXP3+ was found either in OIE or in adult AIE (Tables 3 and 4). No clonal rearrangement of T cell receptor gamma was found in intestinal mucosa of patients treated with olmesartan, while two out of four adult AIE displayed either an oligoclonal (patient 9) or clonal (patient 11) repertoire [5,6].

### Outcome of olmesartan-induced enteropathy (Table 5)

**Table 5:** Treatments.

	Steroids	AZA/6MP	Anti-TNF-α	Cyclosporin	Rapamycin	FK-506	Rituximab
**Olmesartan**							
**1**	10 m:-/ -	4m:-/-	1 m:-/-	-	2m: +/+	8m: +/+	-
**2**	12m: +/+	6m:-/nd	30 m: +/-	-	-	-	-
**3**	6m: +/ nd	48 m: +/nd	-	-	-	-	-
**4**	2 m:-/nd	-	12m: +/+	-	-	-	-
**5**	1m:-/nd	-	6m: +/-	-	-	-	-
**6**	12m: +/nd	12m: +/nd	12m: +/+	-	-	1m:-/nd	-
**7**	12m: +/+	-	12m: +/+	-	-	-	-
**AIE**							
**8**	2m:-/-	1m:-/-	9m:-/-	36m: +/+	-	-	-
**9**	47m: +/+	114m: +/-	2m:-/-	-	-	-	1m:-/-
**10**	15m:-/ nd	2m:-/nd	10 m: +/-	12m: +/+	-	-	nd
**11**	6m:-/nd	14m: +/+	-	-	-	-	1m:-/-

m: month. AZA: azathiopurin. 6MP: 6 mercaptopurin. clinical response (+ or-) / mucosal effect (+/-)

Patients were treated with olmesartan for 2 to 10 years at a mean dosage of 20mg/day at onset of diarrhea. Following diagnosis of villous atrophy resistant to a gluten free diet, they received steroids and immunosuppressors (Table 5). In all but one (patient 4), steroids and immunosuppressors were introduced before olmesartan discontinuation (Table 5). Treatment with anti-TNF-αantibodies induced clinical response in 5 patients (5/6) with partial villous recovery in 3 patients (3/6). FK-506 and rapamycin were used in patient 1, who had collagenous-like sprue and induced complete clinical and histological recovery with disappearance of the collagenous subepithelial layer. After olmesartan discontinuation, remission was maintained despite withdrawal of immunosuppressive drugs in patients 1, 2, 5 and 6. Complete mucosal recovery was confirmed on control intestinal biopsies performed 2 to 7 months later and these patients remain in clinical remission at latest follow-up, one year after cessation of immunosuppression interruption. Patient 4 had stopped olmesartan six months before admission as hypertension had resolved because of the severe diarrhea. Despite this, diarrhea persisted, with important potassium loss that required intravenous supplementation and prolonged hospitalization. Steroids given intravenously were inefficient but infusion of anti-TNF-αantibodies rapidly induced clinical remission. Mucosal healing was observed after seven months of treatment and no relapse was observed after two years without any immunosuppressive treatment. Patient 7 was already treated with anti-TNF-α at time of olmesartan interruption. Despite olmesartan discontinuation, diarrhea relapsed after anti-TNF-α withdrawal but remission was restored when anti-TNF-αtherapy was resumed. Three months after olmesartan discontinuation, he is still treated with anti-TNF-α. Despite being informed of the risks, patient 3 has not stopped olmesartan and still needs azathioprine to control diarrhea.

## Discussion

During the past three years, several cases of severe enteropathy were described in association with olmesartan [2, 12]. A recently published six year-review of pathology reports of patients investigated in the US showed no association between the use of olmesartan and the histological diagnosis of celiac disease or microscopic colitis, respectively [13] suggesting that this severe gastrointestinal disorder is a rare adverse effect of this angiotensin receptor-blocker. Our observations further suggest that OIE affects predisposed individuals. As already reported by Rubio-Tapia, we observed an increased prevalence of the HLA-DQ2/DQ8 genotype (67%), which predisposes to celiac disease or to type I diabetes. In support of our hypothesis, extra-intestinal autoimmune diseases were found in 3/7 patients.

The olmesartan prodrug is converted within the small bowel into olmesartan medoxomil, the active compound, which binds the angiotensin 2 receptor AT1 [14, 15, 16]. How olmesartan can induce severe inflammation and intestinal damage remains to be elucidated. In keeping with the hypothesis of an immune mechanism, OIE referred to our institution shared striking similarities with AIE. In both situations, severe villous atrophy was associated with glandular apoptotic lesions and increased numbers of intestinal T cells expressing the NK markers CD57 or NKG2C. Antinuclear and anti-AIE-75KD or anti-goblet cells antibodies were found in respectively 80% and 43% of patients. Moreover, all patients with OIE responded to immunosuppressive drugs. Cases of patients 4 and 7 are particularly informative, as anti-TNF-α therapy was necessary to achieve remission of a self-sustaining enteropathy after olmesartan discontinuation. It suggests that olmesartan could trigger immune-mediated enteropathy, a hypothesis in line with the inhibitory effects of the angiotensin receptor blockers on transforming growth factor beta, an immunoregulatory cytokine necessary for gut immune homeostasis [17]. Interestingly, cases of enteropathy related to other angiotensin II receptor inhibitors seem to be much less frequent than OIE [4]. The selective role of olmesartan might be explained by its conversion into the active form in the intestine, its long half-life and its efficacy, 30 fold higher than that of other sartans.

Altogether these data support caution when using olmesartan in patients with an autoimmune background.

## References

[1] Systematic review: Sprue-like enteropathy associated with olmesartan.Ianiro G, Bibbò S, Montalto M, Ricci R, Gasbarrini A, Cammarota G. Aliment PharmacolTher. 2014 7;40(1):16–23. 10.1111/apt.12780 24805127

[2] Rubio-Tapia A, Herman ML, Ludvigsson JF, Kelly DG, Mangan TF, Wu TT, et al Severe spruelikeenteropathy associated with olmesartan. Mayo Clin Proc 2012; 87:732–8 10.1016/j.mayocp.2012.06.003 22728033 PMC3538487

[3] DeGaetani M, Tennyson CA, Lebwohl B, Lewis SK, Abu Daya H, Arguelles-Grande C, et al Villous atrophy and negative celiac serology: a diagnostic and therapeutic dilemma.Am J Gastroenterol. 2013 5;108(5):647–53 10.1038/ajg.2013.45 23644957

[4] Olmesartan-associated enteropathy: results of a national survey. Marthey L, Cadiot G, Seksik P, Pouderoux P, Lacroute J, Skinazi F, et al Aliment PharmacolTher. 2014 11;40(9):1103–9. 10.1111/apt.12937 25199794

[5] Malamut G, Verkarre V, Callens C, Colussi O, Rahmi G, MacIntyre E, et al Enteropathy Associated T cell Lymphoma complicating an Autoimmune Enteropathy. Gastroenterology 2012;142:726–729 10.1053/j.gastro.2011.12.040 22226659

[6] Malamut G, Meresse B, Kaltenbach S, Derrieux C, Verkarre V, Macintyre E, et al Small Intestinal CD4+ T cell lymphoma: a heterogenous entity with common pathological features. J Clin Gastroenterol Hepatol 2014;2014;12:599–608 10.1016/j.cgh.2013.11.028 24316103

[7] Jabado N, Le Deist F, Cant A, De Graeff-Meeders ER, Fasth A, Morgan G, et al Bone marrow transplantation from genetically HLA-nonidentical donors in children with fatal inherited disorders excluding severe combined immunodeficiencies: use of two monoclonal antibodies to prevent graft rejection. Pediatrics 1996;98:420–8. 8784367

[8] Malamut G, Afchain P, Verkarre V, Lecomte T, Amiot A, Damotte D, et al Presentation and long term follow-up of refractory celiac disease: comparison of type I with type II. Gastroenterology 2009;136:81–90. 10.1053/j.gastro.2008.09.069 19014942

[9] Verkarre V, Asnafi V, Lecomte T, Patey Mariaud-de Serre N, Leborgne M, Grosdidier E, et al Refractory celiac sprue is a diffuse gastrointestinal disease. Gut 2003; 52:205–211. 12524401 10.1136/gut.52.2.205 PMC1774980

[10] Cellier C, Patey N, Mauvieux L, Jabri B, Delabesse E, Cervoni JP, et al Abnormal intestinal intraepithelial lymphocytes in refractory sprue. Gastroenterology 1998; 114:471–481. 9496937 10.1016/s0016-5085(98)70530-x

[11] Hmida NB, Ben Ahmed M, Moussa A, Rejeb MB, Said Y, Kourda N, et al Impaired control of effector T cells by regulatory T cells: a clue to loss of oral tolerance and autoimmunity in celiac disease? Am J Gastroenterol.2012 4;107:604–11. 10.1038/ajg.2011.397 22108452

[12] Dreifuss SE, Tomizawa Y, Farber NJ, Davison JM, Sohnen AE. Spruelike enteropathy associated with olmesartan: an unusual case of severe diarrhea. Case Rep Gastrointest Med. 2013; 2013:618071 10.1155/2013/618071 23573432 PMC3610358

[13] Greywoode R, Braunstein ED, Arquelles-Grande C, Green PH, Lebwohl B. Olmesartan, other antihypertensives, and chronic diarrhea among patients undergoing endoscopic procedures: a case-control study. Mayo Clin Proc.2014;89:1239–43 10.1016/j.mayocp.2014.05.012 25023670 PMC4157109

[14] Burnier M, Brunner H.R. Angiotensin II receptor antagonists. Lancet, 2000, 355, 637–645 10696996 10.1016/s0140-6736(99)10365-9

[15] Brunner H, Laeis P. Clinical efficacy of olmesartanmedoxomil. J Hypertens, 2003, 21(suppl 2), S43–S46. 12929907 10.1097/00004872-200305002-00008

[16] Kirch W, Horn B, Schweizer J. Comparison of angiotensin II receptor antagonists. Eur J Clin Invest, 2001, 31, 698–706 11473571 10.1046/j.1365-2362.2001.00871.x

[17] Kagami S, Border WA, Miller DE, Noble NA. Angiotensin II stimulates extracellular matrix protein synthesis through induction of transforming growth factor-beta expression in rat glomerular mesangial cells. J Clin Invest 1994; 93: 2431–7 8200978 10.1172/JCI117251 PMC294451

